# Relationship between expectations regarding aging and productive engagement among community-dwelling older adults: a cross-sectional study

**DOI:** 10.3389/fpubh.2026.1743393

**Published:** 2026-02-27

**Authors:** Shuangshuang Dai, Yijia Zhuo, Siyuan Feng, Xinyue Zhao, Beibei Qiao, Jingjing Wang, Mingli Zhao

**Affiliations:** 1 Department of Anesthesiology, The First Affiliated Hospital of Zhengzhou University, Zhengzhou, China; 2 School of Nursing and Health, Zhengzhou University, Zhengzhou, China; 3 Department of Nursing, Shanghai Fifth People’s Hospital, Affiliated to Fudan University, Shanghai, China; 4 Center of Community-Based Health Research, Fudan University, Shanghai, China

**Keywords:** a cross-sectional study, aging, community-dwelling older adults, expectations regarding aging, productive engagement

## Abstract

**Background:**

This study aims to explore the relationship between expectation regarding aging and productive engagement among community-dwelling older adults.

**Methods:**

From May 2023 to November 2023, we employed a convenience sampling method to conduct a survey among older adults in a community located in southern Minhang District, Shanghai, China. Data collection utilized the General Information Questionnaire, Productive Engagement Scale, and the Aging Expectations Scale (ERA-21). Data analysis was performed using SPSS 22.0.

**Results:**

The community-dwelling older adults exhibited an overall expectation regarding aging score of 42.42 ± 7.36, with subdomain scores of 10.11 ± 2.29 for physical health, 18.37 ± 3.89 for mental health, 6.80 ± 2.30 for cognitive function, and 7.14 ± 2.37 for functional independence. Their productive engagement demonstrated a moderate overall score of 39.84 ± 6.83, comprising family caregiving (39.38 ± 6.55), paid employment (43.90 ± 8.79), and volunteer activities (39.38 ± 6.55). Significant differences in productive engagement were identified across age, marital status, number of children, employment status, living arrangements, income source, monthly income, the number of chronic disease, and activity types (*p* < 0.05), Positive correlations observed between productive engagement and all four aging dimensions (*p* < 0.05). Regression analyses indicated age as a negative predictor (*B* = −1.11, *p* = 0.050), while number of children and the four aging expectation subdomains were significant positive predictors (*B* = 1.705, *p* = 0.004; *B* = 0.555, *p* < 0.001; *B* = 0.438, *p* < 0.001; *B* = 0.675, *p* < 0.005; *B* = 0.399, *p* < 0.001).

**Conclusion:**

The community-dwelling older adults exhibited moderate levels of both expectation regarding aging and productive engagement, demonstrating potential for enhancement. All four dimensions of aging expectations showed positive correlations with productive engagement and demonstrated predictive capacity for its intensity, suggesting that community administrators could develop interventions targeting expectation regarding aging to optimize productive engagement among older adults, strategically leverage the human resources of older adults, and ultimately facilitate the realization of productive aging.

## Introduction

The number of older adults is steadily increasing amid the global trend of population aging. By the end of 2020, China had approximately 260 million individuals aged 60 years and above, accounting for 18.7% of the total population. It is projected that by 2050, the older population in China will peak at 487 million, representing 34.9% of the national population (1). In the context of accelerated population aging, the productive engagement of older adults has emerged as a vital strategy for achieving sustainable development goals and enhancing individual well-being (2). This concept has shifted the discourse on aging from a welfare-based to a development-oriented perspective, recognizing older individuals as valuable human resources rather than societal burdens (3).

Productive engagement is defined as any paid or unpaid activity that generates goods or services with social or economic value (4, 5). Previous studies have demonstrated that such engagement, including unpaid work, promotes healthy aging, reduces the incidence of disability and chronic disease, and enhances physical and psychological well-being, including life satisfaction (6, 7). These benefits contribute to relieving the pressure of aging on healthcare systems. Moreover, productive engagement fosters familial and societal welfare and mitigates fiscal pressures associated with a growing older population (2).

Despite the presence of opportunities, participation rates in organized productive roles remain suboptimal in many urban regions, with an estimated 30–50% of older adults not consistently involved (3). Existing programs are primarily limited to traditional volunteer services, lacking diversification into skill-based or technology-oriented roles that may better reflect the evolving interests and capacities of modern older adults (8). Individual characteristics serve as critical determinants of engagement: those with better physical, cognitive, and functional health are more likely to participate in diverse activities (9–11). The influence of educational attainment remains inconclusive. While higher education may enhance employability due to increased human capital (9, 12), it may also reduce employment necessity by improving retirement security (13, 14). Furthermore, social participation and paid employment exhibit a bidirectional relationship, where the former supports continued occupational involvement (9, 12). Economic necessity [e.g., financial needs (15)] and intergenerational responsibilities [e.g., caregiving and grandparenting (10, 11)] constitute core motivators of productive engagement. Such engagement not only directly promotes healthy aging by lowering disability rates, delaying disease progression, and improving psychosocial well-being and life satisfaction (7), but also yields macro-level societal benefits—such as reducing the burden on healthcare systems and easing financial strain through enhanced family support and the accumulation of social capital. Nevertheless, the psychological and cognitive mechanisms underlying these behaviors remain insufficiently explored.

Expectations Regarding Aging (ERA) refer to older adults’ perceived standards for physical and mental functioning in later life. That is, ERA reflects anticipated benchmarks for healthy aging and embodies individuals’ beliefs about aging and its outcomes (16). The level of ERA is closely associated with active daily engagement, particularly with participation in life-course activities (17). ERA serves as a key driver of active and healthy aging by influencing health-promoting behaviors, healthcare-seeking practices, and preventive health decision-making (18, 19). It is also significantly associated with individuals’ physical function, cognitive abilities, psychological well-being, and social involvement (20–22). Notably, Chon et al. (23) demonstrated the high plasticity of ERA: older adults with positive expectations are more inclined to engage in social activities, and early interventions can facilitate the development of realistic and optimistic views about aging, thereby enhancing the aging experience.

Existing research has largely emphasized the influence of objective factors—such as health status and economic conditions—on productive engagement, with limited attention to psychological and cognitive variables. Furthermore, most studies have adopted unidimensional constructs (e.g., subjective age or general attitudes toward aging) to assess older adults’ perceptions of aging and their associations with health behaviors or volunteering (24). Although informed by lifespan developmental theory (25), this approach does not capture the multidimensional and potentially multidirectional nature of aging perceptions (26, 27). Self-perceptions of aging may differ across physical, psychological, and social functional domains. Additionally, the potential moderating role of cultural context remains insufficiently addressed. To bridge these gaps, the present study introduces the multidimensional construct of “expectations regarding aging” as a key psychological variable to elucidate mechanisms influencing productive engagement among older adults, thus offering new directions for targeted interventions.

### Study aim

To examine the relationship between expectations regarding aging and productive engagement among community-dwelling older adults in China.

### Hypotheses

Research Hypothesis (Hr): There is a significant positive correlation between expectations regarding aging and the level of productive engagement.

Null Hypothesis (Ho): There is no significant correlation between expectations regarding aging and the level of productive engagement.

### Research questions

1. What is the level of expectations regarding aging among older adults in Chinese communities?
2. What is the level of productive engagement among older adults in Chinese communities?
3. What is the relationship between expectations regarding aging and productive engagement?

### Methodology

Guided by a positivist research paradigm (28), this study adopts a quantitative approach, based on the ontological assumption that the constructs of expectations regarding aging and productive engagement exist as objective, measurable realities. Epistemologically, this paradigm posits that knowledge about these phenomena can be acquired through systematic observation and measurement, while axiologically, the research process strives for objectivity and value neutrality. Consequently, a cross-sectional design was employed (29). This design is widely regarded as an appropriate choice for such research because it can efficiently quantify the levels of constructs and examine the correlations among them at a single point in time.

## Methods

### Study design

This study employed a cross-sectional design. Under predetermined inclusion and exclusion criteria, community-dwelling older adults were recruited via convenience sampling from the southern area of Minhang District, Shanghai, between May and November 2023. Data were collected through face-to-face interviews using paper-based questionnaires. Each questionnaire was accompanied by an explanatory letter outlining the study objectives, emphasizing the voluntary nature of participation, and assuring respondents of data confidentiality.

### Sample and sampling technique

Eligible participants were community-dwelling older adults recruited via convenience sampling from a community in the southern part of Minhang District, Shanghai. Inclusion criteria were as follows: (1) age 60 years or older; (2) residence in the community for at least 6 months; (3) current involvement in family caregiving, volunteer activities within the past 4 weeks, or paid employment; and (4) provision of informed consent and willingness to participate. Exclusion criteria included cognitive impairments (e.g., diagnosed dementia such as Alzheimer’s disease), speech or communication disorders, or difficulties in understanding the survey instruments. A total of 500 participants were enrolled.

According to Kendall’s method for sample size estimation (30), the minimum required sample size was set at 5–10 times the number of independent variables. With 43 independent variables and a 20% allowance for non-response or incomplete data, the minimum target sample size was 269. The final sample of 500 participants exceeded this requirement.

### Instruments and data collection

Data collection was conducted using a questionnaire comprising three instruments:

### General information questionnaire

This self-developed questionnaire was designed based on the study objectives and a comprehensive literature review. It included demographic and socioeconomic variables such as gender, age, educational level, marital status, number of children, pre-retirement occupation, current employment status, living arrangements, primary source of income, monthly personal income, type of health insurance coverage, chronic health conditions, and main types of productive activities engaged in, among other relevant information.

### Productive engagement scale

The Productive Engagement Scale was originally developed by the American scholar Larry (31) and subsequently translated, culturally adapted, and validated in Chinese by Dai (32). The finalized Chinese version is a unidimensional instrument comprising nine items, with a total score range of 9–63. It utilizes a 7-point Likert scale (1 = “strongly disagree” to 7 = “strongly agree”), with no reverse-scored items. Higher total scores indicate higher levels of productive engagement. Researchers validated the Productive Engagement Scale using the Rasch model. The results showed that the infit (average) value was 1.00 (0.64 ~ 1.38), and the outfit (average) value was 1.02 (0.59 ~ 1.44). The item reliability of this scale was 0.98, and the separation index was 7.53. The scale-content validity index was 0.89. The Cronbach’s *α* was 0.92.

### Expectations regarding aging-21 (ERA-21)

This study employed the ERA-21 scale, which was initially developed by Sarkisian et al. (33) and subsequently translated and culturally adapted into Chinese by Cheng (34) in 2021. The scale comprises 21 items across four domains: physical health, mental health, cognitive function, and functional independence. The Cronbach’s *α* for each dimension are 0.919, 0.910, 0.845, and 0.793, respectively. Responses are recorded using a 4-point Likert scale ranging from 1 (disagree) to 4 (strongly agree), with items 9 and 18 reverse-coded. Total scores range from 21 to 84, with higher scores indicating more positive expectations regarding aging among community-dwelling older adults. In this study, the scale demonstrated good internal consistency, with a Cronbach’s *α* of 0.840.

### Procedure of the study

During the preparatory phase, a comprehensive review of the relevant literature was conducted. A research proposal was subsequently submitted to the appropriate ethics committee to obtain ethical clearance. Community-dwelling older adults were then invited to participate in the survey. A researcher contacted the Chief Nursing Officer of the Nursing Department in a community in Minhang District, Shanghai, to secure institutional support for data collection. Prior to survey administration, all field investigators underwent standardized training to ensure consistent delivery of introductory scripts and explanations regarding questionnaire completion. The purpose and significance of the survey were communicated to participants, and informed consent was obtained to minimize response bias. Data were collected through on-site distribution and immediate retrieval of paper-based questionnaires. Each participant required approximately 10 min to complete the questionnaire. Data collection was conducted between May and November 2023. Upon collection, questionnaires were sequentially numbered, verified independently by two reviewers, and screened for invalid responses.

### Statistical analysis

All data were entered and analyzed using the Statistical Package for the Social Sciences (SPSS) version 22.0. Statistical significance was determined using an alpha level (*α*) of 0.05, with *p*-values ≤0.05 taken as evidence to reject the null hypothesis (H_0_). Descriptive statistics were used to summarize the demographic data: quantitative variables were presented as means ± standard deviations (SD), and categorical variables as frequencies and percentages. Multicollinearity diagnostics were conducted to assess inter-variable associations. Univariate analysis, independent samples *t*-tests, and analysis of variance (ANOVA) were used to explore the impact of demographic characteristics on productive engagement. Spearman’s correlation analysis was employed to examine relationships between the four dimensions of expectations regarding aging (physical health, mental health, cognitive function, and functional independence) and productive engagement. To mitigate overfitting risk, demographic covariates were included only if associated with productive engagement at *p* < 0.05 in univariate analyses. Multicollinearity was assessed using variance inflation factors (all VIF < 10). Model stability was evaluated through internal validation with a randomly split sample to ensure no substantial decline in explanatory power. Finally, multiple linear regression analysis was conducted to evaluate the influence of the four dimensions of aging expectations on productive engagement.

### Consideration of bias and quality control

To enhance the robustness of this cross-sectional observational study, several measures were implemented to address potential biases and ensure the validity and reliability of the findings. Selection bias was mitigated by establishing clear inclusion and exclusion criteria, and by determining the sample size *a priori* using a standard estimation formula to improve representativeness. Information bias was minimized through standardized, face-to-face questionnaire interviews conducted by uniformly trained and qualified interviewers to ensure consistency, with incomplete or logically inconsistent responses subjected to follow-up verification or exclusion. To account for potential confounding, key demographic and health-related variables that met the pre-specified significance threshold (*p* < 0.05) were measured and included in the analysis, accompanied by multicollinearity diagnostics (e.g., Variance Inflation Factor, VIF) to assess and control for inter-correlations among predictors. Additionally, the model was subjected to a validation test to assess for overfitting. Furthermore, the validity of the study was supported by employing instruments with established content validity and by examining construct validity within the analytical framework.

### Ethical approval

The Life Science Ethics Review Committee of Zhengzhou University granted ethical approval for this study (approval number: ZZUIRB2023-060) in late 2019. All procedures were conducted in accordance with the Declaration of Helsinki. Written informed consent was obtained from all participants after the research objectives and procedures were thoroughly explained. Participants were assured of the confidentiality of their personal information. Their rights to privacy and voluntary participation, including the freedom to withdraw from the study at any time, were explicitly communicated. Formal consent was documented to confirm their voluntary and informed agreement to participate.

## Results

### Sociodemographic characteristics of participants (*N* = 500)

A total of 500 questionnaires were distributed and all were completed and returned, yielding a 100% valid response rate. The sociodemographic characteristics of the community-dwelling older adults are summarized in Table 1. More than half of the participants were aged 60–69 years. The majority were married or cohabiting, while a minority were single, widowed, divorced, or never married. Approximately 43% had attained at least a high school education. Regarding pre-retirement employment, 63% had worked in private enterprises and 41% in state-owned enterprises. At the time of the study, 8% of the participants were still employed.

**Table 1:** Demographic characteristics of the study participants (*n* = 500).

Variable	Category	(*n*)	(%)	Productive engagement score	*t*/*F*	*p*
Gender					0^a^	1.000
	Male	190	38.00	39.84 ± 7.53		
	Female	310	62.00	39.84 ± 6.38		
Age					3.835^a^	<0.001
	60—69	331	66.20	40.67 ± 6.84		
	70—79	169	33.80	38.22 ± 6.53		
Education level					0.984^b^	0.400
	Primary school or below	139	27.80	40.56 ± 7.13		
	Junior high school	145	28.90	39.59 ± 6.57		
	Senior high school/technical school	136	27.20	39.87 ± 6.90		
	College or above	80	16.10	39.00 ± 6.64		
Marital status					2.820^a^	0.006
	Married	440	88.00	40.10 ± 6.97		
	Unmarried (widowed/divorced/single)	60	12.00	37.93 ± 5.37		
Number of children					7.564^b^	<0.001
	0 children	5	1.00	33.60 ± 6.77		
	1 child	315	63.00	39.15 ± 6.11		
	2 or more children	180	36.00	41.22 ± 7.72		
Pre-retirement occupation					0.373^b^	0.689
	Farmer	41	8.20	39.15 ± 4.48		
	Private enterprise employee	252	50.40	39.75 ± 7.14		
	State-owned enterprise employee	207	41.40	40.09 ± 6.85		
Employment status					9.914^b^	<0.001
	Unemployed	105	21.00	40.96 ± 6.17		
	Retired	356	71.20	39.10 ± 6.57		
	Employed	39	7.80	43.64 ± 9.00		
Living arrangements					5.268^b^	<0.001
	Living alone	48	9.60	37.31 ± 6.34		
	Living with spouse only	293	58.60	39.60 ± 7.05		
	Living with children only	92	18.40	40.17 ± 5.52		
	Living with spouse and children	67	13.40	42.24 ± 7.15		
Primary income source					6.339^b^	<0.001
	Pension	391	78.20	39.41 ± 6.71		
	Labor income	36	7.20	44.36 ± 9.30		
	Financial support from children	52	10.40	39.38 ± 4.48		
Monthly income (CNY)	Social security benefits	21	4.20	41.24 ± 6.30		
					4.838^b^	0.003
	<1,000	53	10.60	42.30 ± 6.93		
	1,000–2,999	123	24.60	40.76 ± 7.09		
	3,000–5,000	157	31.40	39.52 ± 7.23		
	>5,000	167	33.40	38.69 ± 5.93		
Health insurance type					0.169^b^	0.845
	Resident medical insurance	182	36.40	40.08 ± 7.19		
	Employee medical insurance	283	56.60	39.71 ± 6.86		
	Provincial medical insurance	35	7.00	39.69 ± 4.29		
Number of chronic diseases					3.853^b^	0.022
	None	189	37.80	40.92 ± 6.70		
	1 chronic disease	197	39.40	39.29 ± 6.32		
	≥2 chronic diseases	114	22.80	39.02 ± 7.67		
Primary activity type					8.936^b^	<0.001
	Family caregiving	426	85.20	39.38 ± 6.55		
	Paid work	42	8.40	43.90 ± 8.79		
	Volunteer activities	32	6.40	39.38 ± 6.55		

a Independent samples *t*-test, *t*.

b One-way ANOVA, *F*.

### Means and standard deviations of study variables

The mean score for expectations regarding aging among community-dwelling older adults was 42.42 ± 7.36. Scores for each of the four dimensions were as follows: physical health, 10.11 ± 2.29; mental health, 18.37 ± 3.89; cognitive function, 6.80 ± 2.30; and functional independence, 7.14 ± 2.37. The mean productive engagement score was 39.84 ± 6.83, indicating a moderate level of engagement. Subdomain scores were as follows: family caregiving, 39.38 ± 6.55; paid work, 43.90 ± 8.79; and volunteer activities, 39.38 ± 6.55.

### Univariate analysis of productive engagement by demographic variables

Univariate analysis revealed statistically significant differences in productive engagement scores across the following variables: age, marital status, number of children, current employment status, living arrangements, primary source of income, monthly personal income, number of chronic diseases, and primary type of productive activity (*p* < 0.05). No statistically significant differences were observed in productive engagement scores across gender, educational attainment, pre-retirement occupation, or type of health insurance (*p* > 0.05), as presented in Table 1.

### Correlation between expectations regarding aging and productive engagement

Correlation analysis indicated significant positive associations between productive engagement and all four dimensions of expectations regarding aging—physical health, mental health, cognitive function, and functional independence (*p* < 0.05), as detailed in Table 2.

**Table 2:** Correlation analysis between expectations regarding aging and productive engagement among community-dwelling older adults.

Variable	Physical health	Mental health	Cognitive function	Functional independence
Productive engagement	0.360**	0.401**	0.399**	0.341**

**p < 0.01.

### Multiple regression analysis of productive engagement among community-dwelling older adults

Multiple regression analysis was conducted to identify the factors influencing productive engagement among community-dwelling older adults. Productive engagement was treated as the dependent variable, while independent variables included those demographic factors that were statistically significant at *p* < 0.05 in the univariate analysis as well as the four dimensions of expectations regarding aging.

Multicollinearity diagnostics were performed using standard criteria [tolerance > 0.1, Variance Inflation Factor (VIF) < 10], and no multicollinearity was detected among the variables. The coding scheme for the variables is presented in Table 3. The final regression model retained ten variables: age (60–69), marital status, number of children, employment status, living arrangement, primary source of income, monthly personal income, number of chronic diseases, primary type of activity, and the four dimensions of expectations regarding aging. Collectively, these variables accounted for 40.4% of the variance in productive engagement. Specifically, age was found to be a negative predictor, whereas the number of children and all four dimensions of aging expectations exhibited positive predictive effects. The regression equation is provided in Table 4 and is expressed as follows:

**Table 3:** Variable coding for regression analysis.

Variable	Coding description
Age	“60–69” = 1, “70–79” = 2
Marital status	“Married” = 1, “Unmarried” = 2
Employment status	Dummy variables with “Retired” as reference: X1 (Unemployed = 1, Retired/Employed = 0), X2 (Employed = 1, Unemployed/Retired = 0)
Living arrangements	Dummy variables with “Living with spouse and children” as reference: X1 (Living alone = 1, others = 0), X2 (Living with children only = 1, others = 0), X3 (Living with spouse only = 1, others = 0)
Primary income source	Dummy variables with “Pension” as reference: X1 (Labor income = 1, others = 0), X2 (Financial support from children = 1, others = 0), X3 (Social security = 1; others = 0)
Primary activity type	Dummy variables with “Paid work” as reference: X1 (Family caregiving = 1, others = 0), X2 (Volunteer activities = 1, others = 0)

**Table 4:** Multivariate regression analysis of factors influencing productive engagement among community-dwelling older adults (*n* = 500).

Variable	*B*	Standard error	Beta	*t*	*p*	Collinearity diagnostics	
						VIF	Tolerance
Constant	21.177	3.222	–	6.573	<0.001	–	–
Age	−1.111	0.587	−0.077	−1.891	0.049	1.336	0.749
Marital status	0.199	1.041	0.009	0.191	0.848	1.979	0.505
Number of children	1.705	0.586	0.124	2.910	0.004	1.468	0.681
Monthly income	−0.578	0.36	−0.084	−1.606	0.109	2.213	0.452
Number of chronic diseases	−0.536	0.327	−0.06	−1.638	0.102	1.080	0.926
Employment status (Ref: retired)							
Unemployed	0.989	1.151	0.059	0.86	0.390	3.800	0.263
Employed	−4.138	2.412	−0.163	−1.715	0.087	7.238	0.138
Living arrangements (Ref: living with spouse and children)							
Living alone	−2.889	1.274	−0.125	−2.267	0.024	2.437	0.41
Living with children only	−0.626	0.972	−0.036	−0.644	0.520	2.452	0.408
Living with spouse only	−0.353	0.783	−0.025	−0.451	0.652	2.569	0.389
Primary income source (Ref: pension)							
Labor income	4.742	2.246	0.18	2.111	0.035	5.829	0.172
Social security	−1.215	1.439	−0.036	−0.844	0.399	1.441	0.694
Financial support from children	−2.682	1.213	−0.12	−2.211	0.027	2.370	0.422
Primary activity type (Ref: paid work)							
Family caregiving	−2.077	1.721	−0.108	−1.207	0.228	6.458	0.155
Volunteer activities	−0.617	1.922	−0.022	−0.321	0.748	3.826	0.261
Aging expectations (4 dimensions)							
Physical health	0.555	0.113	0.186	4.903	<0.001	0.555	0.113
Mental health	0.438	0.069	0.249	6.324	<0.001	0.438	0.069
Cognitive function	0.675	0.117	0.227	5.780	<0.001	0.675	0.117
Functional independence	0.399	0.113	0.139	3.535	<0.001	0.399	0.113

Productive engagement = 21.177–1.111 × Age + 1.705 × Number of Children − 4.138 × Employed − 2.889 × Living Alone + (4.742 × Labor Income − 2.682 × Children Support) + (0.555 × Physical Health + 0.438 × Mental Health + 0.675 × Cognitive Function + 0.399 × Functional Independence).

## Discussion

This study examined the association between expectations regarding aging and productive engagement in community-dwelling older adults. The overall score for aging expectations was moderate, with the highest mean score observed for mental health and the lowest for cognitive function. All four dimensions—physical health, mental health, cognitive function, and functional independence—were positively correlated with productive engagement (*p* < 0.05). Among these, mental health exerted the strongest influence, followed by cognitive function and physical health.

Approximately 66% of participants were aged between 60 and 69 years, and 78% reported receiving pension benefits. The results indicated that productive engagement declined with increasing age, consistent with the findings of Sia et al. (2). Participants aged 60–69 generally exhibited better physical health and were more actively involved in social activities aimed at fulfilling their self-worth. Moreover, individuals financially dependent on their children demonstrated significantly lower levels of productive engagement compared to those receiving pensions. Previous studies have shown that older adults with financial stability are more likely to participate in social activities, facilitated by improved living conditions and more optimistic outlooks on aging (35). In contrast, financial dependence on children often correlates with reduced decision-making autonomy within the family, contributing to diminished productive engagement (36).

Family caregiving emerged as the predominant form of productive engagement, with a mean score of 39.38 ± 6.55. More than 85% of participants reported involvement in caregiving activities such as looking after grandchildren, whereas fewer than 15% engaged in paid work or volunteer activities. This trend reflects the enduring influence of traditional intergenerational support norms in Chinese society (37). Motivated by collectivist values, older adults often prioritize family needs over personal pursuits, and are associated with the predominance of family-centered productive engagement (2, 37). In the Chinese cultural context, the family plays a vital role in the lives of older adults, with Confucian notions of filial piety profoundly influencing intergenerational interactions and support patterns (38, 39). Older adults are not only care recipients but also active care providers (40). They generally regard family caregiving as a form of “productive engagement,” a pattern that reflects the persistence of collectivist values. Previous studies have indicated that approximately 65% of grandparents in urban China are regularly involved in grandchild care. Such intergenerational care not only alleviates the childcare burden of their adult children but also provides older adults with a continued sense of role meaningfulness and purpose in life (41, 42). Studies by Silverstein and Zuo et al. (43) suggest that limiting caregiving duties to no more than 30 h per week can help older adults maintain a balance between their own needs and familial responsibilities. In light of these findings, several policy recommendations are proposed: At the community level, mutual-aid networks and dedicated activity spaces should be established to promote peer support and social engagement, while culturally appropriate programs that integrate social participation alongside family caregiving should be developed. At the family level, intergenerational appreciation for elders’ caregiving roles should be fostered through emotional recognition and shared responsibilities. At the governmental level, childcare support systems should be enhanced through parenting subsidies, expanded public childcare services, and flexible work arrangements for younger parents, thereby alleviating caregiving burdens on the older adults and promoting intergenerational equity. In addition, efforts should be made to promote the culture of “productive engagement in later life,” leveraging media and educational institutions to highlight the diverse roles older adults play within both family and society, thereby fostering a social environment for active aging that honors family responsibilities while encouraging personal development.

The results also showed that over half of the older adults cohabited with a spouse, 9.6% lived alone, and 13.4% lived with both spouse and children. Participants living alone reported significantly lower levels of productive engagement, consistent with the findings of Liu and Lou (44). Qualitative studies (45) have shown that older adults living with family report higher levels of well-being and satisfaction when engaging in productive activities. In contrast, those living alone more frequently report social isolation and reduced confidence in their health, factors that are associated with lower willingness to participate in social activities and lower levels of productive engagement. To support productive aging, it is essential to encourage solitary older adults to engage in meaningful activities and to implement tailored strategies to increase their participation.

In the present study, the mean score for ERA among community-dwelling older adults was 42.42 ± 7.36, indicating a moderate level and lower than the results reported by Zeng et al. (46). This difference may be attributed to variations in participants’ socioeconomic conditions, such as living environments, healthcare accessibility, and the comprehensiveness of social security systems.

Among the four ERA dimensions, mental health scored the highest, consistent with findings by Zhao (17). This dimension reflects expectations related to psychosocial adjustment and social interaction. In this study, 90.4% of participants lived with a spouse and/or children, benefiting from stable family ties and community integration. Such social embeddedness likely mitigates feelings of loneliness and psychological distress, thereby contributing to higher mental health scores. Conversely, cognitive function and physical health received lower scores, corroborating the results of Zeng et al. (46). These findings suggest that community-dwelling older adults tend to prioritize mental well-being over physical or cognitive capacity. While positive psychosocial adaptation may be supported by accessible leisure opportunities, reliable healthcare, and stable living conditions, the inevitable nature of age-related decline—such as memory deterioration and physical degeneration—often fosters negative self-perceptions. The tendency to attribute physical and cognitive limitations to normal aging is associated with the neglect of health maintenance and delayed medical intervention, ultimately accelerating health decline (47).

Future policies should prioritize economic and healthcare support to improve physical health and cognitive function among older adults, while simultaneously fostering a supportive social environment that addresses their mental health expectations. Early interventions, such as meditation, audiovisual exercises, and memory training, may enhance aging-related expectations. Previous studies have demonstrated that expectations regarding aging are highly modifiable (48). Therefore, systematic training programs and public health campaigns should be implemented to cultivate optimistic perspectives on aging, thereby empowering older adults to proactively manage age-related changes and achieve active and healthy aging.

Pearson correlation analysis revealed significant positive associations between productive engagement and all four dimensions of expectations regarding aging (physical health, mental health, cognitive function, and functional independence) among community-dwelling older adults, with mental health exhibiting the strongest correlation. This may be attributed to individuals with higher mental health expectations adopting more optimistic attitudes toward aging. Such optimism is associated with both greater motivation for proactive social participation and higher levels of productive engagement (47). The results of the regression analysis indicated that all four dimensions of expectations regarding aging exerted positive predictive effects on productive engagement. Together with demographic variables, these factors explained 40.4% of the variance in productive engagement, thereby verifying the study’s hypothesis and confirming that expectations regarding aging are significant determinants of productive engagement among older adults in community settings.

Higher aging expectations among community dwelling older adults showed positive correlations with better mental wellbeing, physical health, and cognitive function. They were also linked to more proactive health maintenance and more active social engagement, which are key features of productive aging. In contrast, older adults with lower expectations regarding aging tended to show reduced willingness to socialize, lower levels of social participation, and underuse of social resources. These were associated with lower levels of productive engagement. These findings are consistent with those of Andrews et al. (49), who reported that older adults who maintain high expectations for themselves are more inclined to engage in activities beneficial to their physical and mental well-being. Such participation contributes to enhanced quality of life in later years and facilitates the realization of personal value through productive aging (50). Community-dwelling older adults with higher expectations regarding cognitive function and functional independence often demonstrate superior abilities in activities of daily living, reduced dependency, and a greater capacity for productive engagement. According to expectancy-value theory, an individual’s motivation to engage in productive activities is influenced by both their expectations regarding successful participation and the value they assign to those activities (51).

To promote active aging, the implementation of multi-level strategies is essential. When formulating and implementing relevant strategies it is essential to base them on China’s unique family culture and social structure while recognizing family care as a critical component of productive engagement. A systematic approach should be adopted to develop and expand multi-level participation pathways for older adults spanning from family and community to broader societal engagement. This will establish a cohesive and mutually reinforcing system that supports sustained involvement across different domains of life. At the national level, fostering positive societal perceptions of older adults requires the expansion of inclusive social participation platforms and the reinforcement of policy safeguards that support productive engagement. Policy design should ensure cultural adaptability, supporting older adults in maintaining their family roles while also facilitating their broader social integration and value creation. Community-level initiatives should include the establishment of senior talent hubs equipped with skills-matching systems, allowing older adults to register their expertise and access volunteer or employment opportunities. Within families, it is critical to provide older adults with autonomy and encouragement to strengthen their sense of self-efficacy. Additionally, leveraging media and educational institutions to normalize and promote elder participation in productive activities will help establish an age-inclusive social ethos, thereby advancing sustainable and equitable pathways toward active aging.

### Limitations

This study employed a cross-sectional observational design, which helps reveal associations between variables but requires the following limitations to be considered when interpreting and generalizing the results. First, while multiple methods were adopted to minimize common method bias and confounding bias, the cross-sectional design cannot infer causality between expectations regarding aging and productive engagement. The observed associations may reflect reverse causation or confounding effects from unmeasured variables. Furthermore, cross-sectional studies can only describe states at a single point in time and cannot elucidate how these states form, evolve, or develop. Second, although the Chinese version of the Productive Engagement Scale has been validated in urban older populations, its applicability in rural and diverse regional contexts remains to be tested, which may affect the generalizability of the construct measurement. Third, the sample was drawn from only one district in Shanghai, limiting the external validity of the findings to broader socioeconomic and geographic populations across China. Future research should adopt longitudinal or experimental designs to establish causality and include diverse, nationally representative samples to verify and extend the applicability of these findings across different contexts in China.

## Conclusions and recommendations

This study investigated the influence of expectations regarding aging on productive engagement among community-dwelling older adults. The findings indicated that both aging expectations and productive engagement were at moderate levels, suggesting potential for improvement. All four dimensions of aging expectations were positively correlated with productive engagement, and these expectations demonstrated a significant predictive effect, consistent with the study’s hypotheses. Furthermore, productive engagement was influenced by both individual characteristics and aging expectations, with age, number of children, living arrangements, and primary income source identified as key predictive factors.

Based on these findings, it is recommended that community administrators implement targeted interventions to enhance older adults’ aging expectations, thereby improving their productive engagement. Such efforts can contribute to the effective utilization of the older population’s human resources and promote the realization of productive aging.

## Data Availability

The original contributions presented in the study are included in the article/supplementary material, further inquiries can be directed to the corresponding authors.

## Author’s note

JW and MZ are jointly supervised this work.
